# Santos Júnior CJ, Fischer FM. Mental and behavioral disorders related to work in Brazil: temporal trends and the impact of the Social Security Technical Nexus. Cad Saúde Pública 2024; 40(9):e00031524.

**DOI:** 10.1590/0102-311XER031524

**Published:** 2025-05-26

Where it reads:

The increasing trend observed in the incidence rate of work-related mental and behavioral disorders was predominantly attributed to the categories of ICD-10 F40-F48 (APC = 7.293%; p = 0.008) and F60-F69 (APC = 6.95%; p < 0.001). Other categories, such as F00-F09, F10-F19, F20-F29, F30-F39, F50-F59, F70-F79, F80-F89, and F90-F99 did not show statistically significant trends using the employed method (Table 2).

It should read:

The increasing trend observed in the incidence rate of work-related mental and behavioral disorder was predominantly attributed to the ICD-10 category F40-F48 (APC = 7.293%; p = 0.003). Other categories, such as F00-F09, F10-F19, F20-F29, F30-F39, F70-F79, and F90-F99 did not show a statistically significant trend using the employed method (Table 2).

Where it reads:

**Table 2:** Time series and temporal trend of the incidence (per 100,000 links) of work-related mental and behavioral disorders among beneficiaries of the Social Security in Brazil, 2003-2019, according to ICD-10 codes (Chapter V).

Year	F00-F09	F10-F19	F20-F29	F30-F39	F40-F48	F50-F59	F60-F69	F70-F79	F80-F89	F90-F99
2003	0.43	0.25	0.88	6.09	0.06	0.17	0.01	0.04	0.07	0.43
2004	0.41	0.19	1.21	8.87	0.05	0.11	0.02	0.02	0.06	0.41
2005	0.27	0.17	1.63	12.25	0.06	0.15	0.04	0.05	0.07	0.27
2006	0.15	0.17	1.45	10.41	0.04	0.1	0.02	0.01	0.06	0.15
2007	0.79	0.37	11.12	17.47	0.05	0.14	0.02	0.05	0.09	0.79
2008	1.79	1.06	18.13	25.3	0.04	0.18	0.02	0.04	0.16	1.79
2009	1.82	1.21	20.06	27.55	0.09	0.13	0.02	0.03	0.13	1.82
2010	1.53	1.03	15.82	23.8	0.05	0.1	0.01	0.03	0.08	1.53
2011	1.29	0.78	14.27	24.2	0.07	0.11	0.04	0.04	0.09	1.29
2012	1.15	0.65	12.53	26.26	0.04	0.06	0.02	0.01	0.07	1.15
2013	1.05	0.52	13.33	29.71	0.04	0.1	0.02	0.02	0.13	1.05
2014	0.95	0.5	12.19	29.34	0.05	0.07	0.01	0.03	0.1	0.95
2015	0.66	0.38	9.89	33.21	0.07	0.06	0.01	0.03	0.08	0.66
2016	0.56	0.41	9.95	35.05	0.05	0.08	0.01	0.02	0.19	0.56
2017	0.46	0.32	9.03	32.3	0.04	0.04	0.02	0.03	0.07	0.46
2018	0.48	0.34	9.76	29.71	0.04	0.05	0.02	0.04	0.02	0.48
2019	0.44	0.24	11.19	25.75	0.03	0.06	0.01	0.02	0.03	0.44
μ	0.84	0.51	10.14	23.37	0.05	0.1	0.02	0.03	0.09	0.84
APC (%)	-2.92	0.76	0.22	16.08	9.29	-2.12	-6.95	-2.11	-1.7	-3.21
95%CI	-8.33; 2.81	-11.42; 14.61	-11.61; 13.63	-1.01; 36.11	2.69; 16.30	-4.72; 0.54	-8.88; -4.98	-6.15; 2.10	-4.63; 1.32	-11.25; 5.57
p-value	0.288	0.902	0.971	0.064	0.008	0.11	0	0.297	0.246	0.436
Trend	Stationary	Stationary	Stationary	Stationary	Increasing	Stationary	Decreasing	Stationary	Stationary	Stationary

95%CI: 95% confidence interval; μ: mean; APC: annual percent change.

Note: ICD-10 codes (Chapter V): F00-F09 - Organic mental disorders, including symptomatic ones; F10-F19 - Mental and behavioral disorders due to psychoactive substance use; F20-F29 - Schizophrenia, schizotypal, and delusional disorders; F30-F39 - Mood disorders; F40-F49 - Neurotic, stress-related, and somatoform disorders; F50-F59 - Behavioral syndromes associated with physiological dysfunctions and physical factors; F60-F69 - Personality disorders and adult behavior disorders; F70-F79 - Intellectual disabilities; F80-F89 - Disorders of psychological development; F90-F99 - Behavioral and emotional disorders appearing during childhood or adolescence.

It should read:

**Table 2:** Time series and temporal trend of the incidence (per 100,000 links) of work-related mental and behavioral disorders among beneficiaries of the Social Security in Brazil, 2003-2019, according to ICD-10 codes (Chapter V).

Year	F00-F09	F10-F19	F20-F29	F30-F39	F40-F48	F50-F59	F60-F69	F70-F79	F80-F89	F90-F99
2003	0.25	0.37	0.21	0.74	5.16	0.05	0.14	0.01	0.03	0.06
2004	0.20	0.33	0.16	0.99	7.25	0.04	0.09	0.02	0.02	0.05
2005	0.21	0.21	0.13	1.27	9.54	0.05	0.12	0.03	0.04	0.05
2006	0.21	0.12	0.13	1.11	7.96	0.03	0.08	0.01	0.01	0.04
2007	0.53	0.58	0.27	8.10	12.72	0.03	0.10	0.01	0.03	0.07
2008	0.45	1.22	0.73	12.43	17.35	0.03	0.12	0.02	0.02	0.11
2009	0.32	1.10	0.74	12.17	16.71	0.05	0.08	0.01	0.02	0.08
2010	0.21	0.94	0.63	9.67	14.54	0.03	0.06	0.01	0.02	0.05
2011	0.19	0.79	0.48	8.80	14.92	0.04	0.07	0.02	0.02	0.06
2012	0.19	0.72	0.40	7.81	16.37	0.02	0.04	0.01	0.01	0.05
2013	0.15	0.65	0.32	8.18	18.23	0.02	0.06	0.01	0.01	0.08
2014	0.20	0.59	0.31	7.49	18.03	0.03	0.04	0.01	0.02	0.06
2015	0.16	0.41	0.24	6.14	20.60	0.04	0.04	0.01	0.02	0.05
2016	0.15	0.34	0.25	6.13	21.59	0.03	0.05	0.01	0.01	0.12
2017	0.18	0.29	0.21	5.77	20.64	0.03	0.03	0.02	0.02	0.05
2018	0.18	0.31	0.22	6.26	19.05	0.02	0.03	0.02	0.02	0.01
2019	0.11	0.28	0.15	7.16	16.47	0.02	0.04	0.01	0.01	0.02
μ	0.23	0.54	0.33	6.48	15.13	0.03	0.07	0.01	0.02	0.06
APC (%)	-4.579	-0.896	-1.419	13.888	7.293	-3.706	-8.588	-3.792	-3.420	-4.767
95%CI	-9.223; 0.302	-11.665; 11.185	-11.872; 10.273	-1.458; 31.623	2.839; 11.940	-5.686; -1.685	-10.159; -6.989	-7.802; 0.392	-6.479; -0.261	-11.754; 2.773
p-value	0.064	0.870	0.789	0.075	0.003	0.001	0.000	0.072	0.036	0.192
Trend	Stationary	Stationary	Stationary	Stationary	Increasing	Decreasing	Decreasing	Stationary	Decreasing	Stationary

95%CI: 95% confidence interval; μ: mean; APC: annual percent change.

Note: ICD-10 codes (Chapter V): F00-F09 - Organic mental disorders, including symptomatic ones; F10-F19 - Mental and behavioral disorders due to psychoactive substance use; F20-F29 - Schizophrenia, schizotypal, and delusional disorders; F30-F39 - Mood disorders; F40-F49 - Neurotic, stress-related, and somatoform disorders; F50-F59 - Behavioral syndromes associated with physiological dysfunctions and physical factors; F60-F69 - Personality disorders and adult behavior disorders; F70-F79 - Intellectual disabilities; F80-F89 - Disorders of psychological development; F90-F99 - Behavioral and emotional disorders appearing during childhood or adolescence.

